# The efficacy and safety of different negative-pressure wound therapy gradients on flaps outcomes

**DOI:** 10.1007/s13304-025-02156-7

**Published:** 2025-03-23

**Authors:** O. H. Elbanna, A. Salah Eldine, A. M. Sayed, A. K. Mousa

**Affiliations:** 1 Department of Plastic & Reconstructive Surgery, Aswan Faculty of Medicine, Aswan University Hospitals, Kasr Elhagar St., P Box 81513, Aswan, Egypt; 2 Department of Plastic & Reconstructive Surgery, Aswan Faculty of Medicine, Aswan University Hospitals, Aswan, Egypt; 3 Department of Plastic & Reconstructive Surgery, Ain Shams Faculty of Medicine, Cairo, Egypt

**Keywords:** Flaps, Negative pressure wound therapy, Reconstructive surgery, Vacuum-assisted closure, Negative pressure gradients

## Abstract

Negative pressure wound therapy (NPWT) has been shown to be beneficial for improving the wound healing process and reducing flap complications. However, the ideal NPWT settings, especially the pressure levels and application modes, are still debatable. This study examines the efficacy and safety of NPWT at different pressure gradients, namely, high (HNPWT) and low (LNPWT), to determine the optimal conditions for improving flap outcomes and minimizing complications. Over a 30-month period, 65 patients who underwent flap reconstruction were randomly assigned to three groups: HNPWT (75–125 mmHg, continuous mode), LNPWT (50–75 mmHg, intermittent mode), and conventional wound dressing (CWD). Patients were evaluated prospectively for post-operative complications, flap viability, infection, edema, and wound dehiscence. Complications were more common in the CWD group than in the HNPWT group, while the HNPWT group  had the highest incidence of flap ischemia (41%). NPWT significantly reduced post-operative edema (*P* = 0.003) and lower infection and dehiscence rates than the CWD group (*P* = 0.015 and *P* = 0.029, respectively). Compared with HNPWT, LNPWT showed superior safety and efficacy, with fewer ischemic events, lower pain scores, faster wound healing times, and better esthetic and function outcomes. Although NPWT offers benefits over conventional wound dressing in flap reconstructions, pressure settings should be carefully adjusted. LNPWT is safer and has more satisfactory outcomes than HNPWT, with reduced ischemia and better overall healing. These findings suggest that LNPWT in the intermittent mode is most favorable for improving flap viability and minimizing adverse effects.

*Registration identification number* NCT06080958- July 22, 2024. “Retrospectively registered” URL for the registry: http://www.clinicaltrials.gov/

## Introduction

Flaps are a fundamental reconstructive option in plastic and reconstructive surgery and employed to address complex tissue defects resulting from trauma, surgery, or congenital abnormalities. These versatile tissue transfers involve the transplantation of vascularized tissue from one site of the body to another, ensuring the transfer of not only skin but also underlying fat, muscle, or bone as necessary. Compared with other reconstructive techniques, flaps provide superior outcomes as they offer improved wound coverage, enhanced functional restoration, and esthetic benefits. However, the use of flaps is not without its challenges and potential complications. The complications associated with flap procedures include but are not limited to ischemia, necrosis, infection, hematoma, seroma formation, and impaired wound healing [1, 2].

NPWT has evolved as a revolutionary approach in wound management. Its history dates back to the early 1990s. NPWT involves the application of subatmospheric pressure to a wound through a sealed dressing connected to a vacuum source. The mechanisms of action underlying NPWT are multimodal and include improved tissue perfusion, reduced  edema, promotion of angiogenesis, and enhanced granulation tissue formation. Negative pressure facilitates the removal of excess interstitial fluid, decreases bacterial colonization, and promotes the formation of a healthy wound bed. NPWT has diverse applications across various medical specialties, including the management of chronic wounds, surgical incisions, and traumatic injuries. Its utility extends to both acute and chronic wounds, demonstrating its effectiveness in enhancing tissue viability and expediting the overall wound healing process. As a result, NPWT has become a valuable tool in the armamentarium of clinicians, contributing to improved patient outcomes in the field of wound care [3].

The history of NPWT in the context of flap salvage dates back to the early 2000s, when clinicians began to explore its potential as an adjunctive tool  for optimizing outcomes  of compromised flaps. Prior to the widespread adoption of NPWT in flap salvage, traditional methods rely on dressings and surgical interventions to manage complications, such as compromised blood supply, infection, and poor tissue perfusion in flaps were implemented [4].

One of the pivotal moments in the integration of NPWT into flap salvage occurred when clinicians recognized its ability to create a controlled and optimized wound environment. The application of negative pressure helps reduce edema, increase blood flow, and promote tissue perfusion, creating favorable conditions for flap survival. Studies and case reports in the mid-2000s began to highlight the positive impact of NPWT on salvaging compromised flaps, particularly in cases where traditional methods  had limitations [3, 5].

Adjustable NPWT represents a recent advancement in wound care technology that allows healthcare professionals to tailor negative pressure settings according to individual patient needs. The ability to modify the key parameters influences the efficacy, safety, and overall usage of NPWT in wound management. The adjustable parameters of NPWT are integral to tailoring treatment strategies to individual patients and wound characteristics. These adjustable parameters include negative pressure levels, cycle types (continous versus intermittent), cycle times, dressing types and sizes, pressure alarms, and adherence monitoring. The negative pressure levels can be optimized to enhance tissue perfusion and manage patient discomfort. Adjustable cycles influence the frequency of wound debridement, facilitating a cleaner wound bed. The choice between continuous and intermittent therapy allows for flexibility on the basis of the wound type and healing stage. The versatility of dressing types and sizes accommodates various wound sizes and shapes. Collectively, these adjustable parameters empower healthcare professionals to customize NPWT, contributing to improved patient outcomes and the successful management of diverse wound scenarios [6].

This study presents a novel understanding of the optimization of negative-pressure wound therapy settings, specifically through systematic evaluation of different pressure gradients (high and low) and application modes (continuous versus intermittent) for flap reconstruction. Most previous studies on NPWT have broadly explored its benefits for various wound types, but few have focused on its precise, controlled use in flap-based reconstructive surgeries where tissue viability and complication rates are critical. This study is among the first to directly compare high and low negative pressures on flap viability and complication outcomes, offering data-driven guidance to clinicians regarding safer, more effective NPWT practices tailored to flap reconstructions.

## Patients and methods

This prospective, randomized, comparative clinical study was carried out from May 2021 to December 2023. All adult patients between the ages of 16 and 60 who presented with post-traumatic or post-tumor excision soft tissue defects, reconstructed  using either a fasciocutaneous or muscle flap, were included. Patients presenting with uncontrolled chronic diseases were excluded from the study.

The included patients were randomly allocated into three groups via the card shuffling method (the patient was asked to pick up a card or piece of paper that determined the study group to which he would be admitted). All wounds were assessed by a modified TIME-H scoring system to ensure the adequacy of the wound bed for reconstruction [7]. In high negative-pressure wound therapy (HNPWT), the negative pressure ranges between 75 and 125 mmHg in continuous mode. In low negative-pressure wound therapy (LNPWT), the negative pressure ranges between 50 and 75 mmHg in intermittent mode (five minutes active and two minutes off). For  the conventional wound dressing (CWD) group, the suture line was cleaned, and the antibiotic ointment, Fusidic acid, was applied once daily.

Prophylactic intravenous amoxicillin/clavulanic acid was administered at a dosage of 1.2 g every 12 h to all patients in the perioperative and post-operative settings. All patients received 40 units of low molecular weight heparin (Clexan) intraoperatively, which was continued for ten days postoperatively. A simple stitch or stapler was used for wound closure every 1 cm.

For patients receiving NPWT, (Medvac system) with a foam dressing was used. The dressing was applied intraoperatively via a sterilized setup. The foam was applied over the flap edges at a suture line of 1–2 cm on each side, sparing the flap pedicle area with a central observation window to monitor skin color and temperature. The machine was adjusted to deliver negative pressure measured at the wound surface from 75 to 125 mmHg in the HNPWT group and from 50 to less than 75 mmHg in the LNPWT group. The NPWT dressing was changed every 3 days and discontinued after the third dressing.

In all patients, we used a rubber drain or tube drain with a distal end embedded in the foam of the NPWT or connected to a low-suction system. The drain was removed during the first NPWT dressing after three days.

Patients were discharged from the hospital on the third postoperative day—when possible—after their first dressing. The patients were then followed up in the outpatient clinic on the sixth and ninth days, weekly for two months, and then monthly for six months. At each visit, the flap vascularity was monitored by temperature, capillary refill, and handheld Doppler. The degree of edema was evaluated on a pitting scale on the third day postoperatively, where 1–2 mm is considered normal, 3–4 mm is mild, 5–6 mm is moderate, and more than 7 mm is severe. Additionally, the degree of pain was self-assessed by all patients via the visual analog scale (VAS), with zero  indicating very dissatisfied and ten indicating very satisfied.

The primary outcomes were post-operative complications, such as congestion, ischemia, infection, or wound dehiscence. Secondary outcomes included the  degree of edema, pain score, and time for complete wound healing.

Long-term esthetic outcomes were evaluated using the Patient and Observer Scar Assessment Scale (POSAS) at the 6-month follow-up visit. This validated tool includes separate scales for the clinician and the patient, assessing parameters, such as vascularity, pigmentation, thickness, pliability, pain, itching, and overall scar appearance. Each parameter was scored on a scale of 1–10, with lower scores indicating scars closer to normal skin [8].

All data were analyzed using  the Statistical Package for the Social Sciences (SPSS 21). Continuous variables are summarized as means ± standard deviations, while  categorical variables are presented as frequencies and percentages. One-way ANOVA was  used to compare measurable variables if the data were  normally distributed; otherwise,  the Kruskal‒Wallis test was  used. The chi-square test will be used for categorical variables. If any expected cell count was  less than 5, Fisher’s exact test was  used. P -values less than 0.05 were  considered statistically significant.

### Power calculation

A power analysis was performed to determine the required sample size for detecting a significant difference in the incidence of distal flap ischemia between the groups. Based on  pilot data, we anticipated an ischemia rate of 42% in the HNPWT group and 0% in the LNPWT group. Using a two-tailed test with a significance level (α) of 0.05 and 80% power, the required sample size was calculated to be 11 patients per group. Given the unequal allocation ratio and the different group sizes, we acknowledge that some comparisons may be underpowered, particularly in the HNPWT group. Therefore, additional studies with larger sample sizes are recommended to confirm these findings.

## Results

This study included 65 participants between May 2021 and December 2023. The mean follow-up duration was 6 months, with a minimum of four months.  Assessments were conducted on the 6th and 9th post-operative days, weekly for the first two months, and monthly thereafter. At the two-month follow-up visit, eight patients were lost to follow-up, and were excluded from the trial. We only enrolled 12 patients in the HNPWT group due to the higher  rate of complications in this group. Meanwhile,  17 patients were treated with CWD, 23 patients were included in the LNPWT group.

The mean age of all patients was 24.9 ± 6.8 years. Seventy-two percent of the participants were males. Trauma was the major cause of soft tissue defects (75%),  and almost half of the defects were located in the lower limbs. There was no statistically significant difference in patient demographics between the groups. All patient demographics are tabulated in **Table **1.

**Table 1:** Demographics and patients characteristics

		HNPWT *N* = 12	LNPWT *N* = 23	CWD *N* = 17	*P* Value
Age (Years)		24.8 ± 6.4	26.3 ± 7.1	26.7 ± 7.5	0.472 ^**(K)**^
Gender	Male	11	18	13	0.669 ^**(F)**^
	Female	1	5	4	
Cause Of Defect	Trauma	9	18	12	0.831 ^**(F)**^
	Tumor Ablation	1	2	1	
	Electrical Burn	0	2	1	
	Pressure Ulcer	2	1	3	
Site of defect	Head & neck	1	2	2	0.595 ^**(F)**^
	Upper limb	5	8	3	
	Trunk	2	1	3	
	Lower limb	4	12	9	
Smoking	Yes	9	16	10	0.697 ^**(F)**^
	No	3	7	7	
Flap Pedicle	Pedicled	12	18	14	0.285 ^**(F)**^
	Free	0	5	3	
Type Of Flap	Radial Forearm	1	2	3	0.945 ^**(F)**^
	Lateral Arm	1	3	1	
	Gluteus Maximus	2	1	2	
	Tensor Fascia Lata	0	0	1	
	ALT	1	3	2	
	Gastrocnemious	2	3	1	
	Reversed Sural	1	4	4	
	Propeller	2	2	0	
	Random	2	5	3	

(k) Kruskal–Wallis test

(c) Fisher’s Exact Test

**Figure 1:**
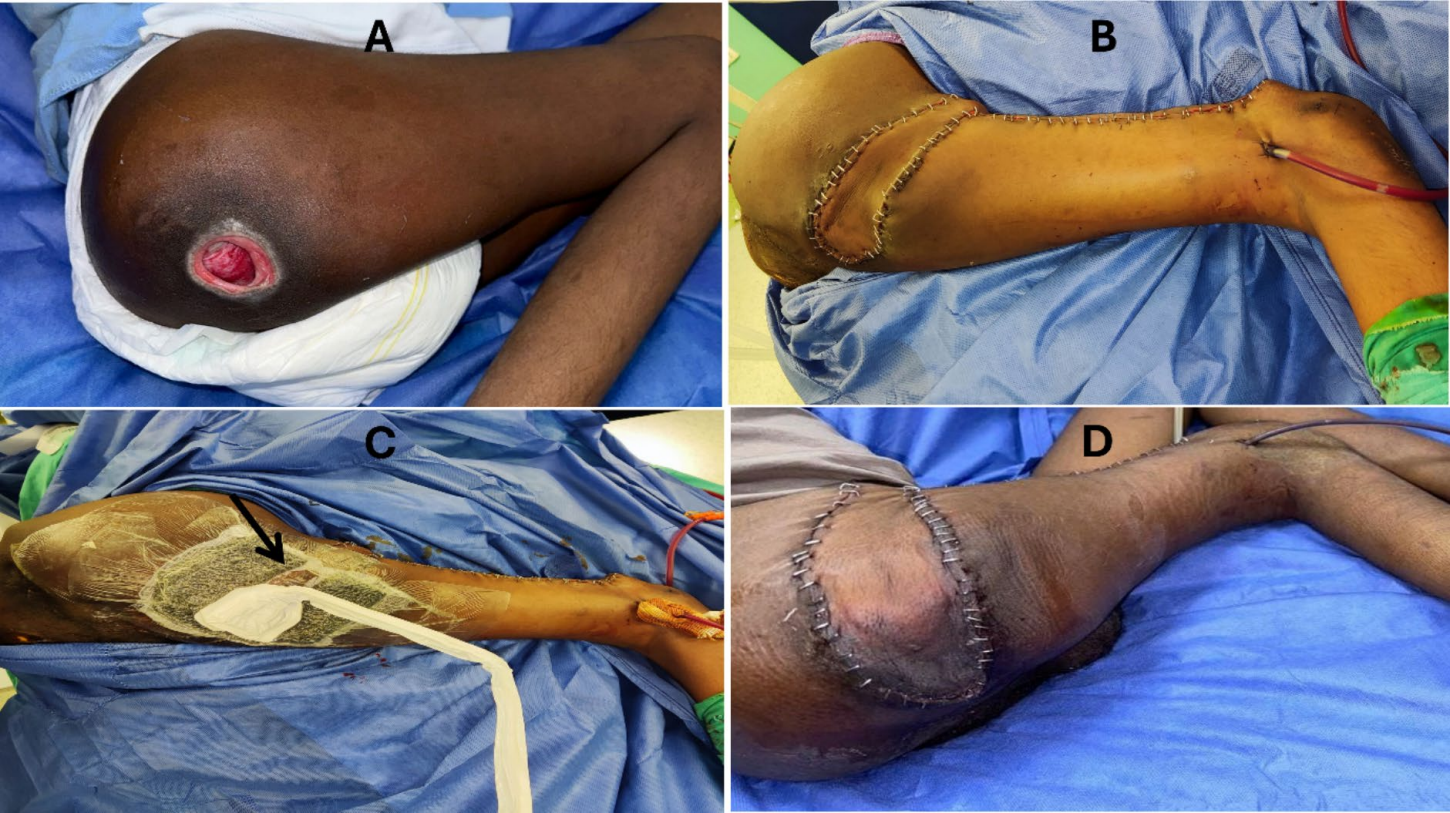
A 42-year-old paraplegic male patient presented with a right ischial pressure ulcer reconstructed with a pedicled ALT flap. **A** Picture of the ischial pressure ulcer. **B** Reconstruction via a pedicled ALT flap. **C** Application of HNPWT at 125 mmHg in continuous mode; the black arrow points to the central part of the flap, which is covered only by  opsite and connecting tubes. **D** Distal flap ischemia under the VAC dressing

Although the overall prevalence of complications was higher in the CWD group (70%) than in the HNPWT and LNPWT groups (41% and 13%, respectively), complications were more critical in the HNPWT group. Compared with 1 patient (5.9%) in the CWD group and 0% in the LNPWT group, five patients in the HNPWT group (42%) presented with distal ischemia, with a highly significant difference between the groups (*P* = 0.001) (Figure. 1).

**Figure 2:**
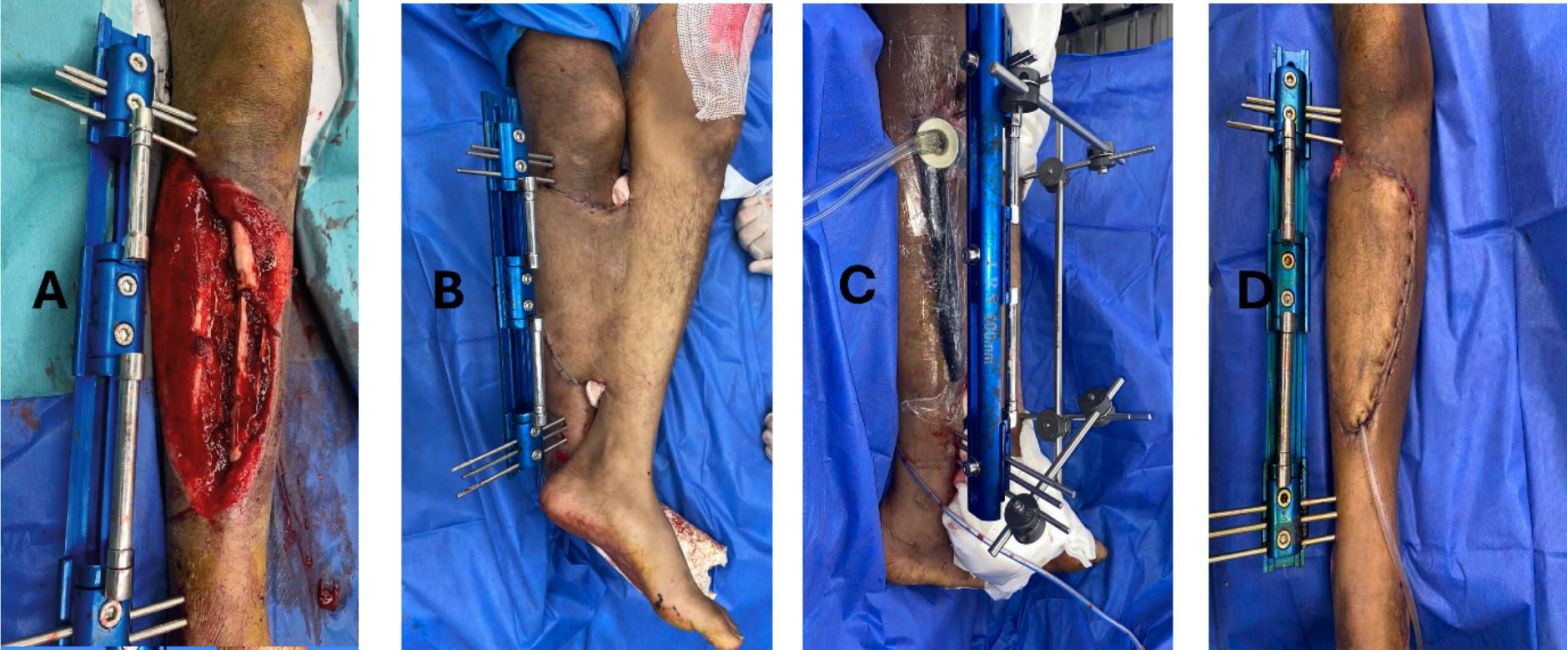
A 33-year-old male patient presented with a post-motor car comminuted tibial fracture with soft tissue loss in the upper and middle thirds of the right lower limbs. The defect was  reconstructed via a cross-leg flap. **A** Soft tissue defect with both the tibia and fibula exposed. **B** Picture after reconstruction via a cross-leg flap. **C** Application of LNPWT at a pressure of 60 mmHg in intermittent mode. **D** Picture of the flap after separation

On the other hand, both HNPWT and LNPWT  appeared to be protective against wound infection compared with CWD (0% and 4.3% vs. 30%, respectively; *P* = 0.015). Additionally, wound dehiscence was significantly lower in both HNPWT and LNPWT than in CWD (0% and 0% vs. 18%, respectively; *P* = 0.029).  Although the incidence of flap congestion was lower in HNPWT and LNPWT than in CWD, this difference was not statistically significant (0% and 8.7% vs. 18%, respectively; *P* = 0.175) (Figure 2).

Compared to  the conventional dressing group, the degree of edema was considerably lower in both the HNPWT and LNPWT groups (*P* = 0.003). Also, the results revealed a statistically significant difference in the mean pain score between LNPWT on one hand and HNPWT and CWD on the other hand (3.9 ± 1.6 vs. 5.2 ± 1.2 and 5.8 ± 1.7, respectively; *P* = 0.002). Furthermore, the healing time in the LNPWT group (9.5 ± 3.2 days) was significantly shorter than in the HNPWT group (17.6 ± 5.2 days) and the CWD group (12.7 ± 5.5 days) (*P* = 0.001). All results are tabulated in **Table** 2.

**Table 2:** Outcomes and complications of all patients

		HNPWT *N* = 12	LNPWT *N* = 23	CWD *N* = 17	*P* Value
Infection	Yes	0 (0%)	1 (4.3%)	5 (29.4%)	0.027 ^**(F)**^
	No	12 (100%)	22 (95.7%)	12 (70.6)	
Congestion	Yes	0 (0%)	2 (8.7%)	3 (17.6%)	0.349 ^**(F)**^
	No	12 (100%)	21 (91.3%)	14 (82.4%)	
Ischemia	Yes	5 (41.7%)	0 (0%)	1 (5.9%)	0.001 ^**(F)**^
	No	7 (58.3%)	23 (100%)	16 (94.1)	
Wound Dehiscence	Yes	0 (0%)	0 (0%)	3 (17.6%)	0.041 ^**(F)**^
	No	12 (100%)	23 (100%)	14 (82.4%)	
Degree of Edema (on Day 3)	Grade 1 (1–2 M)	3 (25%)	6 (26.1)	0 (0%)	0.009 ^**(F)**^
	Grade 2 (3–4)	6 (50%)	13 (56.5)	5 (29.4)	
	Grade 3 (5–6)	3 (25%)	4 (17.4%)	9 (52.9%)	
	Grade 4 (7–8)	0 (0%)	0 (0%)	3 (17.6%)	
Healing Time (Days)		17.6 ± 5.2	9.5 ± 3.2	12.7 ± 5.5	0.000 ^**(K)**^
Pain Score		5.2 ± 1.2	3.9 ± 1.6	5.8 ± 1.7	0.006 ^**(K)**^
Range of motion	Normal	6 (41.7%)	18 (78.3%)	11 (47.1%)	0.037 ^**(F)**^
	Slightly limited	4 (33.3%)	5 (21.7%)	5 (41.2%)	
	Severely limited	2 (25%)	0 (0%)	1 (11.8%)	
Patient satisfaction		5.7 ± 1.5	8 ± 0.85	6.6 ± 0.93	0.000 ^**(K)**^

(k) Kruskal–Wallis test

(c) Fisher’s Exact Test

In addition to early post-operative outcomes, we evaluated long-term esthetic and functional results. The cosmetic appearance of the flap and scar formation were assessed using the Patient and Observer Scar Assessment Scale (POSAS) at the 6-month follow-up visit. As shown in **Table** 3, the LNPWT group demonstrated significantly better scar appearance and higher patient satisfaction compared to the HNPWT and CWD groups (*P* < 0.05). Functional recovery was assessed using range of motion (ROM) and patient-reported outcomes for flaps involving joints or functional areas. Patients in the LNPWT group reported better mobility, reduced pain, and higher  overall satisfaction compared to the other groups.

**Table 3:** Scar Assessment Using the Patient and Observer Scar Assessment Scale (POSAS)

Parameter		HNPWT *N* = 12	LNPWT *N* = 23	CWD *N* = 17	*P* Value
Observer Scale	Vascularity	3.5 ± 0.7	4.0 ± 0.9	4.8 ± 1.0	0.035^**(K)**^
	Pigmentation	3.5 ± 1.3	4.2 ± 1.1	5.1 ± 1.2	0.048^**(K)**^
	Thickness	3.8 ± 0.8	4.5 ± 1.0	5.2 ± 1.1	0.015^**(K)**^
	Relief	3.2 ± 0.7	3.8 ± 0.8	4.5 ± 0.9	0.02^**(K)**^
	Pliability	5.7 ± 1.0	6.5 ± 1.3	7.2 ± 1.4	0.03^**(K)**^
	Surface Area	4.8 ± 0.9	5.5 ± 1.1	6.2 ± 1.3	0.025^**(K)**^
	Overall Opinion	4.08 ± 1.24	4.75 ± 1.38	5.5 ± 1.48	0.000^**(K)**^
Patient Scale	Pain	6.0 ± 1.2	7.0 ± 1.5	7.8 ± 1.6	0.01^**(K)**^
	Itching	4.2 ± 0.9	5.0 ± 1.2	5.8 ± 1.1	0.04^**(K)**^
	Color	5.9 ± 1.2	6.8 ± 1.4	7.5 ± 1.5	0.018^**(K)**^
	Stiffness	7.0 ± 1.5	8.0 ± 1.8	9.0 ± 2.0	0.008^**(K)**^
	Thickness	4.5 ± 1.9	7.1 ± 2.3	9.3 ± 2.8	0.07^**(K)**^
	Irregularity	6.5 ± 1.3	7.5 ± 1.6	8.5 ± 1.8	0.009^**(K)**^
	Overall Opinion	5.68 ± 1.59	6.9 ± 1.84	7.98 ± 2.12	0.000^**(K)**^

(k) Kruskal–Wallis test

## Discussion

It is widely accepted that the majority of open wounds, either acute or chronic, are successfully managed with NPWT [9]. The main advantages of NPWT are as follows: (a) The semipermeable dressing maintains a moist and warm environment for healing; (b) The pressure gradient between the wound and the suction canister reduces wound edema by draining excess fluid from the wound bed and interstitial space; (c) Wound deformation leads to skin graft or flap apposition to the wound bed and approximation of the wound edges together; (d) The risk of wound dehiscence is decreased by reducing lateral strain at the suture site; and (e)  NPWT promote wound healing by increasing the blood supply and minimizing the bacterial load, thereby reducing tissue inflammation [10, 11].

Despite these benefits, many questions remain concerning the application of NPWT to flaps. The two most frequent concerns are the inability to clinically monitor the transferred tissue and the potential for flap compression due to the device's subatmospheric pressure, which could result in flap loss and vascular compromise [12].

Our findings support previous reports that LNPWT is associated with a significantly lower frequency of flap ischemia [13]. However, applying HNPWT (higher than −75 mmHg) in continuous mode may lead to disastrous results. Five flaps (42%) in the HNPWT group exhibited distal ischemia. The findings of Borgquist et al. [14] may help explain this phenomenon. They observed a drop in subcutaneous tissue microvascular blood flow 0.5 cm from the wound margin. For a negative pressure of −80 mmHg, they calculated a 97% decrease in capillary blood flow. Concurrently, they described an increase in perfusion 2.5 cm from the wound edge. This is explained by an increase in the velocity of red blood cells. Biermann et al. [15] upon examining the pressure distribution in the foam's three-dimensional profile, they discovered that the pressure intensity was far greater near the foam's edge than in the center, beneath the trackpad.

On the other hand, a lower incidence of flap ischemia, ranging from 10 to 25%, has been reported in other studies [4, 5, 11, 16, 17]. Most of  these studies are retrospective and  involve a wide range of negative pressures (75–125 mm Hg). Others made some modifications, such as delaying HNPWT insertion for several days or using an intermittent mode  to maintain adequate tissue perfusion between negative cycles. Additionally, all these studies reported the negative pressure that was adjusted on the machine, which is greater than the actual pressure delivered to the wound. This discrepancy could explain the lower incidence of complications in these studies.

Our research confirms earlier findings that the infection rate is substantially lower in the NPWT groups. The transparent plastic coverings of the NPWT create a septic area by isolating the flap from the external environment. Furthermore, low pressure effectively removes wound exudate, inhibiting bacterial overgrowth [5, 17–19]. Additionally, the mechanical force applied by NPWT  significantly decreases tension throughout the wound and aids in approximating the wound edges. The resistance of sutures that received NPWT to separation was 51% greater than that of sutures alone [3]. This tension release not only lowers the chance of wound dehiscence but also facilitates quicker and more efficient healing [5, 19, 20].

The discomfort experienced after removing the dressing for the first time can be reduced by applying NPWT to the flap suture margins [21]. Our findings demonstrate that during the first dressing change, the NPWT group experienced  significantly less pain on the visual analog scale (VAS)  compared to the CWD group. NPWT allows for delayed, and less frequent dressing changes, which further reduces patient discomfort. Usually, the patient feels pain during NPWT at the beginning of each cycle, which makes the continuous mode less painful than the intermittent mode. However, this was  not occur here, likely due to the use of relatively low negative pressure and  modern machines that gradually  decrease the pressure. Similar  results  have been noted in other studies [5, 14, 22]. Compared  to that in the CWD group, edema was noticeably lower in the NPWT group. Flaps, especially island or free flaps, tend to have a bulky appearance, leading to lower overall esthetic satisfaction among patients, and  sometimes necessitating  revision or debulking procedures [23]. Chim et al. [13] noted that considerable edema reduction has been observed with NPWT, which consequently improves  tissue perfusion. Compared to  standard dressings, NPWT has been demonstrated to reduce flap edema, flap thickness, and improve cosmetic outcomes.

We noticed that the degree of edema was less at  the flap edge, (i.e., the area under the foam), than at the flap center. Additionally, the amount of drained fluid in the negative-pressure canister was minimal in almost all cases. We  hypothesize that the reduction in flap edema is not solely due to fluid draining through the wound edge but rather  that NPWT acts as a lymphatic massage, facilitating extracellular fluid drainage through the lymphatic system.

Our findings suggest that NPWT, particularly LNPWT, not only reduces early complications but also improves long-term esthetic and functional outcomes. The reduced scar formation and improved tissue pliability observed in the LNPWT group may be attributed to the controlled pressure and intermittent mode, which promote better tissue perfusion and healing. Also, the reduction  in  postoperative flap edema and the improved scar quality and tissue pliability in the NPWT groups help maintain a near-normal range of motion across the affected joints compared to the CWD  group.

This study lays a foundation for future research aimed at refining NPWT parameters for tailored patient care. Addressing gaps in the understanding of the specific effects of pressure levels and application modes encourages  further trials to investigate detailed NPWT adjustments across various flap types and wound scenarios. We also hypothesize that applying the NPWT dressing over the entire  flap in intermittent mode could improve postoperative results and further reduce  edema. This is considered another research point for further assessment.

Another promising area for future research is the combination of NPWT with advanced wound dressings or wound care technologies. Bioactive dressings, antimicrobial dressings, hydrogels, collagen-based materials, and hyperoxidized oils-based medical devices [24] have shown significant potential in enhancing wound healing. When used in conjunction with NPWT, these dressings could further reduce infection rates, improve tissue hydration, and promote angiogenesis. Future studies could explore the synergistic effects of NPWT and advanced dressings, particularly in complex wound scenarios. For instance, silver nanoparticle containing dressings or wound irrigation with antiseptic solutions could be combined with NPWT to enhance infection control, while hydrogels could be used to reduce pain during dressing changes. Such innovations could expand the scope of NPWT applications and improve patient outcomes.

Furthermore, the role of advanced technology in modern Plastic Surgery cannot be ignored. Emerging tools, such as virtual wound measuring systems, 3D imaging, hyperspectral imaging, and laser scanning; allow for better preoperative assessment, adequate planning, and reliable postoperative monitoring. For example, hyperspectral imaging can provide real-time data on tissue oxygenation and perfusion, while 3D imaging can track changes in wound volume and contour over time [25]. These technologies could be used in future studies to provide a more objective and comprehensive evaluation of flap viability and wound healing.

In our study, we compared two different degrees of negative pressure in two different modes with CWD. Although many authors recommend the use of high negative pressure (− 75 to – 125 mmHg) in continuous mode, we prove that low negative pressure (− 50 to – 75 mmHg) is as effective as high negative pressure and safer. We believe that the use of NPWT results in better esthetic and functional outcomes  and is more cost-effective than secondary surgeries. Therefore, we recommend using LNPWT in intermittent mode unless there is a graft; we recommend continuous mode to avoid shear  movement.

Our study has several limitations. The small sample size and the lack of multicenter data may affect the generalizability of our results. Additionally, we fail to attribute the adverse  effect in the HNPWT group to either the high negative pressure itself, the continuous mode, or both.

## Conclusion

Although NPWT has better results than CWD, it should be used with caution to avoid complications. The results of LNPWT are comparable to those of HNPWT, but LNPWT is safer and has a lower incidence of complications.

## Data Availability

The datasets generated and analyzed during the current study are available from the corresponding author upon reasonable request
